# Giving cell phones to pregnant women and improving services may increase primary health facility utilization: a case–control study of a Nigerian project

**DOI:** 10.1186/1742-4755-11-8

**Published:** 2014-01-20

**Authors:** Sunday Oluwafemi Oyeyemi, Rolf Wynn

**Affiliations:** 1 Department of Clinical Medicine, University of Tromsø, UNN-Asgard, N-9291 Tromsø, Norway; 2 Division of Addictions and Specialized Psychiatric Services, University Hospital of North Norway, Tromsø, Norway

**Keywords:** Maternal health, Health service utilization, Cell phone, Abiye project, Nigeria

## Abstract

**Background:**

Worldwide, about 287 000 women die each year from mostly preventable complications related to pregnancy and childbirth. A disproportionately high number of these deaths occur in sub-Saharan Africa. The Abiye (‘Safe Motherhood’) project in the Ifedore Local Government Area (LGA) of Ondo-State of Nigeria aimed at improving facility utilization and maternal health through the use of cell phones and generally improved health care services for pregnant women, including Health Rangers, renovated Health Centres, and improved means of transportation.

**Methods:**

A one-year sample of retrospective data was collected from hospital records and patients’ case files from Ifedore (the project area) and Idanre (control area) and was analyzed to determine healthcare facility utilization rates in each location. Semi-structured questionnaires were used to generate supplemental data.

**Results:**

The total facility utilization rate of pregnant women was significantly higher in Ifedore than in Idanre. The facility utilization rate of the primary health care centres was significantly higher in Ifedore than in Idanre. The number of recorded cases of the five major causes of maternal death in the two LGAs was not significantly different, possibly because the project was new.

**Conclusions:**

Giving cell phones to pregnant women and generally improving services could increase their utilization of the primary healthcare system.

## Background

Worldwide, about 287 000 women die each year from mostly preventable complications related to pregnancy and childbirth [1-3]. More than 95 per cent of these deaths occur in sub-Saharan Africa and Southern Asia [3-5], which prompted the United Nations into listing ‘Improving Maternal Health’ as the 5th Millennium Development Goal. The target was to reduce maternal deaths by three quarters by 2015 [2,3]. It is now a year to the end of the time frame and many countries are showing worrying signs of failure to achieve this goal [6]. Africa bears the highest burden of maternal deaths in the world [2]. Nigeria has about 2% of the world’s population, while accounting for close to 10% of the world’s maternal deaths [4,7,8].

A majority of the deaths related to pregnancy and childbirth can be prevented if the women receive adequate and timely medical care at the crucial moments [5,9-16]. Evidence suggests that providing expectant mothers with adequate maternal care, birth supervision by skilled attendants, and access to emergency obstetric care in pregnancy and delivery can save lives [17-19]. The complications that can potentially lead to death exist in about 9-15% of pregnancies [20,21]. 75% of maternal deaths are as a result of direct causes from severe haemorrhage (bleeding), maternal sepsis (infection), obstructed labour, high blood pressure with fits in pregnancy (eclampsia) and unsafe abortion [5,22].

Delays in seeking healthcare, in reaching a facility, and in receiving care at a facility, are important factors accounting for much of the maternal deaths in sub-Saharan Africa [4,7-10]. Delays are due to heterogenic myriads of constraints, including financial, social, cultural, literacy, geographical, and so on [23].

In a national maternal mortality study conducted in Egypt [11], delay in seeking medical care and non-compliance with medical advice by the patient was found to contribute to 42% of all the maternal deaths. This similitude exists in Nigeria, where Chukudebelu and Ozumba [24] found that delays or unbooked obstetric emergencies (that is, unregistered pregnancies coming in emergency situations) accounted for about 70% of the maternal deaths. Most of these women failed to receive antenatal care, and/or arrived at the health facility moribund or when life was already endangered by difficult labour, advanced pregnancy complications, or concurrent diseases. In a study from rural Kenya, 64% of pregnant women who wanted to deliver in the hospital were unable to reach the hospital in time [25].

It has been estimated that 70% of the population of sub-Saharan Africa now have access to mobile phones [26-28], with very good cellular telephone signals already present in almost all the remote and poor communities [26,29,30]. This technology might be used in combatting maternal mortality [10,27,31-34]. However, few have systematically examined the effects of use of cell phones on clinical outcomes in low-income countries [35,36], and little is known about whether this technology may help in improving maternal health [27,32,36]. While many maternal deaths are multi-factorial [9], cell phones could help in improving services and reducing delays, for instance by making it easier for pregnant women to get needed information and help, and increasing deliveries under trained personnel, by giving health workers increased access to expert advice and improved possibilities for requesting help or referring patients to better equipped centres when complications occur [37-44]. Pregnant women seem to be positive to mHealth interventions [34,45]. Following improved communications through a radio system, Samai and Sengeh [41] found that the number of pregnant women with major obstetric complications arriving at the hospital from the project area increased from 0.9 to 2.6 per month. Furthermore, the case fatality rate dropped from 20 percent to 10 percent. Matthews and Walley [40] found that giving walkie-talkies to project-trained traditional birth attendants increased facility utilization by the pregnant women.

The Abiye project was launched in Ondo State, Nigeria, in October 2009, with the objective to increase facility utilization, reduce maternal deaths, and reduce neonatal mortality. Prior to this, the state was reportedly having the highest maternal death rate in south western Nigeria as stated by the World Bank and quoted by the State Government on the State website [46,47]. A baseline survey carried out by the State Government just before the commencement of the Abiye project found that only 16% of pregnant women who registered at the health facilities eventually delivered their babies there [46,47].

The project involved 17 health facilities: 1 General Hospital, 2 Comprehensive Health Centres and 14 Basic Health Centres in Ifedore Local Government Area, Ondo State, Nigeria. Pregnant women contacting any of the Abiye designated health facilities for the first time, were registered, assigned a Health Ranger, and given free, Closed-Users’ Group (CUG) cell phones, which were also given to health workers in the project area. Through these phones, the women could communicate free of charge with the healthcare services and among themselves. The goals were to increase access to the health services, increase health facility utilization, and reduce missed medical appointments. It was also expected that the system would allow for express communication with the healthcare team in times of emergency, better access to family planning information, and ultimately abating the series of inherent delays and hence reducing maternal deaths in the LGA.

Health Rangers are specially trained Community Health Extension Workers residing in the local communities in the Abiye project area. Their training includes, but is not limited to, basic obstetric care, intensive care, expanded life saving skills and family planning. Each Health Ranger is assigned 25 registered pregnant women whom she is expected to track and monitor regularly with a customised checklist. In order to evacuate patients and mobilise the Health Ranger as required, appropriate means of transportation suitable for the LGA of operation were provided in form of motorcycles, tricycles and four-wheel drive ambulances. The Basic Health Centres and the Comprehensive Health Centres in the LGA were renovated and drugs, consumables and other necessary materials were provided. The services were made free of charge to the pregnant women.

The purposes of this study were to compare the facility utilization rate of the pregnant women participating in the Abiye project in Ifedore LGA to those of a control group in neighboring Idanre LGA. We also wanted to compare the frequency of occurrence of the 5 major causes of maternal deaths in the project area and the control area. An additional purpose was to identify the benefits and challenges of the Abiye cell phones to the users, as reported in a questionnaire.

## Methods

This was a case–control study where retrospective data were collected from patients’ case files and hospital records in the 10 participating health facilities in the 2 LGAs. These data included the total number of antenatal (pregnancy) registrations and baby deliveries at the facilities. The facility utilization rate was determined by the ratio of the number of deliveries in a particular health facility to the number of antenatal clinic registrations in that same facility, within a specified period of time. Data on the frequency of occurrence of the 5 major causes of maternal death (severe bleeding, maternal infection, eclampsia, obstructed labour, and unsafe abortion) for each month of the year 2011 were also collected. All pregnant women who attended the health facilities in the period 1 January – 31 December 2011 were included in the study.

However, the following were excluded from the study: 1) Women whose records had shown medical conditions that could interfere with understanding and following instructions or significant cognitive impairment. 2) Women with severe psychiatric disorders. 3) Women with major cardiovascular disease. 4) Women with sickle cell disease or other haemoglobinopathies.

Three types of health facilities were included in the study: 1) Basic Health Centres, which are basic healthcare units existing in almost all the small villages and towns. They provide routine medical care for common ailments mostly free of charge or at a nominal cost. The Basic Health Centres also run family planning care, maternal and child health services. 2) The Comprehensive Health Centres are bigger, better equipped and staffed than the Basic Health Centres and perform basic laboratory services, collection and reporting of vital statistics, simple surgical interventions, caesarean sections and referral services. 3) The 15 General Hospitals are about evenly distributed all over the State with one in each LGA, while the State Specialist Hospitals exist in the major towns of each of the three senatorial districts of the State. The fourth and the most equipped, well-staffed of the Specialist Hospitals is located in Akure, the capital of the state. This capital is about equi-distance to the 2 LGAs where this study was carried out.

The study was conducted in Ondo-State, one of the 36 states in the Federal Republic of Nigeria. It is located in the south-western region of the country with a population of about 3.44 million people. The State is divided into 3 senatorial districts, which are further divided into 18 Local Government Areas (LGAs). The research sites were chosen from 2 LGAs: Ifedore LGA (population 176 000) and Idanre LGA (population 129 000). Ifedore LGA was chosen because that was where the Abiye project was on-going and Idanre LGA was used as a comparison because it was a nearby and largely similar to Ifedore LGA in socio-economy and healthcare arrangements, apart from the Abiye project. The neighbor area (Idanre LGA) was chosen as a control area as we assumed that all the pregnant women in the project area (Ifedore LGA) could be exposed to the project in some way.

The project (case) data were retrospectively collected from five health centres from Ifedore LGA that were participating in the Abiye Program (General Hospital Igbara-Oke; Comprehensive Health Centre, Ijare; Comprehensive Health Centre, Ilara Mokin; Basic Health Centre, Isarun; and Basic Health Centre, Molete). The control data were collected from five health centres from the neighboring Idanre LGA (General Hospital, Idanre; Comprehensive Health Centre, Owena; Basic Health Centre, Ofosu; Basic Health Centre, Ese-Oke; and Basic Health Centre, Alade Atosin). Thus, both the General Hospitals in the LGAs were included and the Comprehensive Health Centres selected in Ifedore LGA were the 2 centres where Abiye programme was on-going, while the 2 centres chosen in Idanre LGA were selected randomly – as were the 4 Basic Health Centres used in the study (i.e. two from Abiye participants and two from the Idanre LGA).

It is time-consuming and expensive to measure maternal mortality in developing countries like Nigeria, where reliable records are not available [48]. Therefore, in lieu of measuring maternal mortality, this study made use of the 5 major causes of maternal deaths, sometimes referred to as “the big five” [49,50], namely:

1. Severe bleeding (antepartum, intrapartum or postpartum haemorrhage that necessitated hospital admission or referral).

2. Hypertensive disorder of pregnancy with fits (antepartum, intrapartum or postpartum eclampsia with or without documented preeclampsia necessitating hospital admission, or referred as a result).

3. Infection (maternal sepsis that warranted hospital admission and parenteral antibiotic administration, or referred to another centre).

4. Obstructed labour (resulting from any cause, ensuing onto a caesarean section, with or without a live baby, or referred as a result).

5. Unsafe abortion (or its complications, in the guise of illegal abortion, septic abortion, incomplete abortion, admitted into the hospital, or referred).

The link between the Abiye program and the five major causes of maternal death is as follows: If the program is successful in making pregnant women make use of the health services in a timely manner, it may be possible to either prevent the medical or obstetric conditions from occurring, or detecting the illnesses before they end fatally. For instance, a pregnant woman with pre-eclampsia (high blood pressure, foot swelling, and protein in the urine) could be monitored closely and thus eclampsia (a cause of death) could be prevented from occurring.

The main research objectives were: 1) To compare facility utilization rate of the pregnant women participating in the Abiye project in Ifedore LGA to those of a control group in neighboring Idanre LGA. 2) To compare the frequency of occurrence of the 5 major causes of maternal deaths in the project area and the control area. 3) To identify the benefits and challenges of the Abiye cell phones to the users, as reported in a questionnaire.

Drawing on prior studies as well as on the expressed aims of the Abiye project, we have the following two hypotheses:

1) The primary healthcare utilization rate of pregnant women was higher in the project area than in the control area.

2) The frequency of occurrence of the 5 major causes of maternal deaths in the project area was lower than in the control area.

The data (number of registered pregnant women, number of deliveries, number of patients suffering from the five major causes of maternal death) were retrospectively collected from each of the 10 included health facilities for the whole year of 2011. These numbers were used to calculate utilization rates (number of deliveries divided by number of registrations) for each facility individually, for the primary health care centres in the project and control area, and total utilization rates for both areas. T-tests were used to compare the facility utilization rates of the primary health care centres and the secondary health care centre in each area, to compare the facility utilization rates of the primary health care centres in both areas, to compare the facility utilization rates of the secondary health care centres in both areas, and to compare overall facility utilization rates in both areas. The number of patients suffering from the five major causes of maternal death were summed up from the five participating health facilities in each of the areas, and the total numbers of the two areas were compared by means of an odds ratio calculation.

Semi-structured questionnaires were also used to collect data from pregnant women at the antenatal hospital visits in the selected 5 health facilities in Ifedore LGA. This was largely a different sample than those who participated in the case–control study, as the questionnaires were distributed during the period 2–20 July, 2012 (and the case–control data were sampled from hospital records from 2011). The questionnaire consisted of questions related to the index pregnancy, the Abiye cell phone, and Abiye project itself. It was used to assess the users’ perceptions of benefits of the cell phone, effects on health facility utilization, the wish for the continuity of the cell phone initiative, among other topics. The questionnaires were in both English and Yoruba (the local language). Women who visited the facilities for antenatal and postnatal hospital visits in this period were given the questionnaires and were explained the purpose of the study both in writing (i.e. this was written on the questionnaires) and orally and participated voluntarily and anonymously. No names, addresses or other identifying information was collected with the questionnaires, and individual respondents were untraceable. The data from the questionnaires were presented through absolute numbers (n) and percentages.

The permission to carry out all parts of this project, including the questionnaire –study, was obtained from the North Norway Regional Committee for Medical and Health Research Ethics (REK North) and from the Health Research Ethics Committee of the Federal Medical Centre at Owo in Ondo-State, Nigeria. Official authorization and clearance to use the hospital data was also obtained from the State Ministry of Health and the LGA headquarters in Ifedore and Idanre. All the patient data were collected anonymously.

## Results

### Facility utilization

The total number of registrations at the included healthcare facilities was 1429 in Ifedore LGA and 1801 in Idanre LGA. The total number of deliveries at the facilities was 620 in Ifedore and 660 in Idanre (Tables 1 and 2). In Ifedore LGA, the facility utilization rates of the primary health care centres (i.e. Basic and Comprehensive Health facilities) were 43.6%, 51.0%, 45.5% and 71.6%, or 54.4% combined (Table 1). That of the secondary healthcare facility (i.e. the General Hospital) was 31.6%. The facility utilization rate was significantly higher in the primary health care centres than in the secondary healthcare facility (t-test, t(1427) = 8.690, p < 0.001). In Idanre LGA, the facility utilization rates of the primary health care centres were 31.8%, 26.3%, 25.6% and 37.4%, or 30.6% combined (Table 1). That of the secondary healthcare facility was 40.9%. The facility utilization rate was significantly higher in the secondary healthcare facility than in the primary health care centres (t-test, t(1799) = 4.464, p < 0.001). When comparing the facility utilization rates of the primary health care centres in the two LGAs, the rate in Ifedore was significantly higher than in Idanre (t-test, t(1478) = 9.261, p < 0.001). The total facility utilization rates including all 10 centres was significantly higher in Ifedore (43.4%) than in Idanre (36.7%, t-test, t(3228) = 3.866, p = 0.0001).

**Table 1:** Facility utilization and illness cases in project area (Ifedore LGA) in 2011

**Health centre**	**Ante-natal registrations (no.)**	**Deliveries (no.)**	**Facility utilization (%)**	**Illness cases**^**1 **^**(no.)**
GH Igbara	690	218	31.6	12
CHC Ijare	225	98	43.6	6
CHC Ilara	255	130	51.0	5
BHC Molete	215	154	71.6	0
BHC Isarun	44	20	45.5	0
Total	1429	620	43.4	23

^1^The five major causes of maternal death (combined).

**Table 2:** Facility utilization and illness cases in control area (Idanre LGA) in 2011

**Health centre**	**Ante-natal registrations (no.)**	**Deliveries (no.)**	**Facility utilization (%)**	**Illness cases**^**1 **^**(no.)**
GH Idanre	1060	433	40.9	18
CHC Owena	66	21	31.8	4
CHC Ofosu	80	21	26.3	4
BHC Ese-Oke	317	81	25.6	2
BHC Alade Atosin	278	104	37.4	1
Total	1801	660	36.7	29

^1^The five major causes of maternal death (combined).

### Prevalence of the 5 major causes of maternal death

The number of recorded cases of all of the five major causes of maternal death in the project and the control areas were 23 and 29 respectively (Tables 1 and 2). The difference was not statistically significant (OR =1). There were no recorded cases of complications of unsafe abortion in the two study areas in 2011.

### Questionnaire responses

250 questionnaires were given out and 231 were returned (92.4% response rate). 112 (50%) had received an Abiye cell phone. All stated that they actively used the phone. In addition, 92 of those who had an Abiye cell phone also had a private cell phone, while this was the case with 60 of those who did not have an Abiye phone. Thus, 23.5 percent did not have any cell phone at all. Out of the population of the pregnant women using the cell phone, 77% were using it to make calls only, and less than one quarter (23%) were using it for both calls and text messages. Most (95%) of the calls and text messages were made to the health services and only a few (5%) to fellow pregnant women. The phones were used mostly at the last 3 months of pregnancy, with 35 percent making more than 20 calls per month.

Almost all the pregnant women (98%) believed that the number of women dying in pregnancy or shortly after delivery had been reduced drastically, and when asked how they knew this, more than 70 percent stated there was no longer news or information about women’s death in pregnancy in the community since the program commenced. The majority (81%) rated the Abiye cell phone “very useful”. 59% found the easy and free communication linkage with the health workers the most important benefit of the cell phone, while 32% valued more the prompt response to emergency situations afforded by the cell phone. 15% stated the cell phone had improved their use of the health facility. Only 7% considered the cost saving benefit of the cell phone more important. 80% of the women were “mostly satisfied” with the response they got each time they called or texted the health workers or the facility, while 20 percent were “sometimes satisfied”. Less than one quarter (23 percent) of the pregnant women were using the cell phone for family planning information.

The reported barriers to the use of Abiye cell phones were especially related to the infrastructure. 36% cited electricity to charge the phone as a challenge, whereas 27% cited network failure as the major issue. 37% did not report any challenges to the use.

49.1% walked to their local health facility, 33.6% used a motorbike, and 17.2% traveled by car or bus.

## Discussion

The main findings in this study were: 1) Total facility utilization rate (the ratio of the number of deliveries to the number of antenatal registrations) was significantly higher in the project area (Ifedore LGA) than in the control area (Idanre LGA). 2) The facility utilization rate of the primary healthcare facilities was significantly higher in the project area than in the control area. 3) There was no significant difference in the rates of the five major causes of maternal mortality in the project area and the control area.

### Facility utilization

The number of ante-natal registrations was somewhat higher in the control area (Idanre) than in the project area (Ifedore), while the number of deliveries in the control area was only slightly higher than in the project area (Tables 1 and 2). We do not know why more women registered in Idanre than in Ifedore, but it could possibly be related to geographical or socio-demographic factors. Nevertheless, it is possible that this finding could be of importance to the delivery rate, as a relatively high number of registrations possibly could impact the level of resources available and thereby the number of women who chose or were able to deliver at the healthcare facilities in Idanre.

In the project area, the primary healthcare facilities had higher facility utilization rates than the secondary healthcare facility, while the reverse was the case in the control area. In addition, the total facility utilization rate was higher in the project area. One reason for these differences may be that – through the use of the cell phones- the pregnant women in the project area were better acquainted with the local primary healthcare services and thereby might be more confident that they would receive the care they needed when delivering. The primary healthcare facilities (Basic Health Centres - BHC and Comprehensive Health Centres - CHC) were linked with the secondary healthcare facility (General Hospital - GH), in the sense that the BHC and CHC referred patients to the GH and could receive feedback from the GH. Moreover, the lesser trained (in the BHC and CHC) could communicate with and receive support from the experts (in the GH), which could have boosted the confidence of those working in the primary healthcare facilities. The availability of free cell-phones in the project area, and in the project area healthcare facilities, could possibly also have enhanced communication between the primary healthcare facilities and the secondary healthcare facility. Thereby, fewer patients and health workers felt the need to visit (or recommend) the General Hospital. An important consequence would be that the General Hospital in the project area would avoid congestion from cases manageable at the primary healthcare levels and that this could free up resources for cases that should be managed on the secondary level. This means that the higher rate of secondary healthcare facility use in the control area could, in part, be due to referrals that had not been necessary if they had had better means of communication (i.e. more phones) available. The implication of this is that the Abiye cell phones may have strengthened the primary healthcare system in the LGA and have reduced inequalities in accessibility to healthcare facilities, and by extension, cell phone usage may be said to have been a strengthening factor in the primary healthcare delivery system.

### Rates of five major causes of maternal mortality

There was no significant difference in the rate of the five major causes of maternal mortality in the project area and the control area. Various reasons could be responsible for this. First, it might have been too early to detect changes in rates of these illnesses as the program had been in place only about one year [51]. Second, the sample size might have been too small to detect any differences. However, there was a limit to this size because it was a pilot program in relatively rural communities. Third, the program might not have an effect on the rates of the five major causes of maternal mortality. It could be noted that the occurrence of these 5 causes of maternal death does not automatically translate to death. It is the delay in seeking, reaching or receiving timely care at the critical moments [5,9,10] that may result in the death of the pregnant women.

### Use and satisfaction with the Abiye project

The pregnant women who had received the Abiye phones used the phones and almost all found it ‘very useful’, which is a prerequisite for the successful implementation of this type of technology in health care [52]. However, only about 50% of the pregnant women in the Abiye project had actually received cell phones. This was due to a very high demand and irregular supplies of cell-phones, resulting in periodic shortages. Adding personal cell phones, as many as 77% of the pregnant women in the Abiye area had access. However, as the personal cell phones were not free of charge, this could potentially impede their use. Alternative strategies not depending on available phones could be to implement toll-free numbers or distribute special SIM-cards for free calls. More than half of the respondents gave easy and free accessibility as the most valued benefit of the cell phones. The most important factor was probably the ability to get help in emergency situations (i.e. from Health Rangers, Health Clinics or ambulances). It is during the last trimester (and the first few weeks after delivery) that the pregnant women in Ifedore LGA make the most phone calls to the health facilities. The high number of calls during this period could be linked to the fact that this is the most critical period of the pregnancy, and that there is a high need for help, advice, education, and reassurance during this period [53] – which the pregnant women could get from their Health Rangers or nurses at the health facilities through the cell phones.

Text-messaging has become an important means of communication among the healthy as well as the ill in many parts of the world [45,54]. Prior studies have suggested that services that utilize texting can be helpful for pregnant women [34,36,38,55]. While we in our study lack information about why only 23% of those who had access to cell-phones used their cell-phones for texting, it could be that low literacy-rates in rural areas in part could explain this finding [56].

The majority of women asked in this study could walk to their nearest health facility in order to deliver the baby, the rest could reach the facility by motor bikes or bus/car. This means that these women had a greater chance of reaching the health facility in time for delivery than what has been found in some other studies from other African countries [25]. While most of the women did not report any problems in using their cell phones, a small minority had experienced network failure or inadequate power supply for charging, which are well known challenges in some parts of rural Africa [41].

### Limitations

We lack baseline data from both LGAs, which means that we cannot be fully certain about the importance of the Abiye project in creating the differences in facility utilization between the two LGAs.

A part of this study was based on data from the hospital case files and records. Any imperfection in the records would be reflected in the study data. Some pregnant women registered at several health facilities, in order to be able to choose between facilities, receive multiple incentives, or even to guard against strike action. This could in some part account for the relatively low utilization rates. However, it may be assumed that this phenomenon would affect all areas similarly. There were no registered cases of unsafe (illegal) abortion in the study. Abortion is illegal in Nigeria except for medical reasons. Nevertheless, unsafe abortion is not uncommon [57,58]. While there might not have been any cases of unsafe abortion in the study area and period, the lack of registered cases might also represent a reporting issue.

While free cell phones were not available in the control area, some of the pregnant women there had personal cell phones, which in this design could lead to an underestimation of the benefits of cell phones. Moreover, as the two areas were neighboring, some pregnant women could cross over between the areas and thereby also contribute to a possible dilution of effects.

## Conclusions

The study supported the hypothesis that giving pregnant women access to free cell phones and generally improving services (through the Abyie project) would increase their utilization of the primary healthcare system. Improved access to health services and increased facility utilization could lead to a reduction in the rates of the five major causes of maternal death, as the preventive and early intervention services offered at the health facilities might help some pregnant women avoid getting affected by these causes such as unsafe abortion or eclampsia. It is possible that this type of effects could occur at a later stage, when the program is even more established, but it was not possible to detect any such effects at this stage. About half of the pregnant women polled had received an Abiye phone, they reported that the phone was very useful, that it was used the most in the critical period of the third trimester, and that getting help in emergencies was one important result of the phone. The study suggests that the use of cell phones could strengthen the primary healthcare system and increase access to healthcare. This is an important public health implication, especially in the rural parts of sub-Saharan Africa in need of reinforcement of the primary healthcare system.

## Competing interests

The authors declare that they have no competing interests.

## Authors’ contributions

SOO designed the study, collected and analyzed the data, made the initial draft, revised the manuscript and approved the final manuscript. RW designed the study, revised the manuscript and approved the final manuscript.
